# Spinocerebellar ataxia 2 in a family with different phenotypes: Two case reports

**DOI:** 10.1097/MD.0000000000017834

**Published:** 2019-11-15

**Authors:** Yuanyuan Li, Ying Chang, Xiufeng Liu, Yanyan Li, Yayun Yan

**Affiliations:** Department of Neurology, China-Japan Union Hospital of Jilin University, China.

**Keywords:** CAG repeat, Parkinsonism gene, phenotype, SCA2

## Abstract

**Rationale:**

Spinocerebellar ataxia 2 (SCA2) is a genetic disease, mainly characterized by ataxia. A number of other neurological symptoms also have been described, such as Parkinsonism, cognitive dysfunction, autonomic dysfunction, even the signs of motor neuron disease and so on. Mostly, In the same family, clinical performance is the same in most cases. Here, we describe a father and his son who suffered from SCA2, but their first manifestations were different.

**Patient concerns:**

The father exhibited progressive bradykinesia and rigidity, which resulted in the dysfunction of walking and caring himself. He hoped to relieve his symptoms by taking medicine. But the son presented with ataxia which was mild that the discomfort did not affect his daily life with none treated.

**Diagnosis:**

Both of them were given SCA2 tests. Briefly, we designed primers around the CAG trinucleotide, repeated the spinal cerebellar ataxia subtype gene, performed PCR expansion, and then calculated the specific number of repetitions by capillary electrophoresis. Abnormal expansion was detected in them through SCA2 sequencing with different repeat numbers of CAG, and then they were diagnosed with SCA2 sequencing.

**Interventions:**

The father was treated with dopaminergic drugs, but the son was not administered treatment.

**Outcomes:**

The father's symptoms are improved and he can take care of himself. The son has none difficulty in his daily life.

**Lessons:**

It is rare that different individuals in the same family with SCA2 have different manifestations. The genetic testing is a crucial method to diagnose the disease of SCA2.

## 1 Introduction

Spinal cerebellar ataxia (SCA), also known as autosomal dominant ataxia, is characteristic of degeneration of cerebellar, brain stem, and spinal cord, whose dominant manifestation is ataxia. There are now at least 43 subtypes genetic ataxias recognized. Spinocerebellar ataxia mutations may lead to classic Parkinson syndrome. The most common subtypes are SCA2, SCA3, and SCA17.^[1]^ SCA2 has been recently recognized as an uncommon cause of Parkinsonism, an alternate presentation to the typical cerebellar disorder.^[2]^ This phenotype appears to be occur preferentially in Asian populations.^[3]^ Whether it is ataxia or Parkinsonism, a family's clinical manifestations are always the same; reports of one family showing different neurological phenotypes are rare. Here, we report a family with SCA2 in which members have different clinical manifestations. The family relationship is father and son (the proband). The first clinical manifestation of the son is ataxia and the father is bradykinesia.

## 2 Clinical material of two cases

### 2.1 Case 1

**Figure 1:**
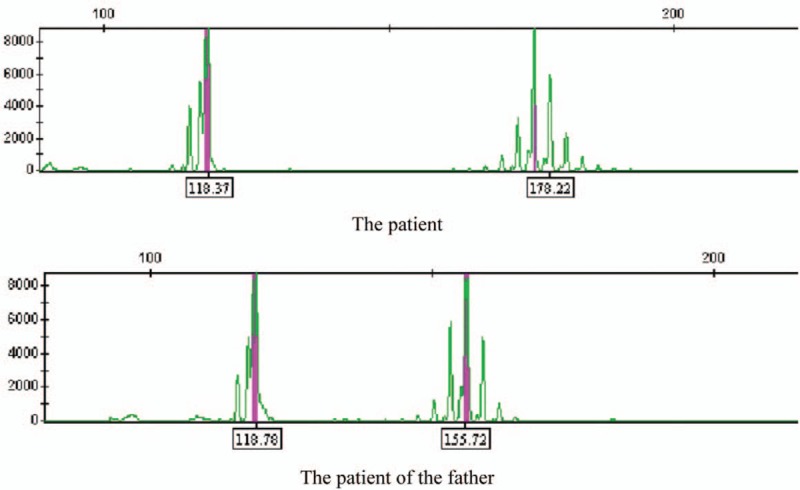
Horizontal axis shows fragment length; vertical axis shows fluorescence signal intensity.

The proband was a 35-year-old male who presented with progressively worsening instability while walking over 5 years. Physical examinations revealed cerebellar ataxia, which were mainly on finger-nose testing, the alternating movement test was uncoordinated, and the Romberg sign positive with eyes closed and opened. Gait was slightly broad based without shuffling. The cranial nerves were normal, as were strength and sensation. He could work and perform daily living activities relatively normally. SCA2 genetic examination revealed expansion to 43 CAG repeats (Table 1, Fig. 1). He can basically live and work normally, so we did not give medication.

**Table 1:**
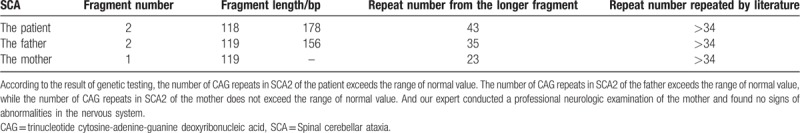
Testing CAG repeats by PCR-STR, the results are as follows.

### 2.2 Case 2

The father of the proband was a 64-year-old male with an onset of bradykinesia. He was very well until he was 60 years old, when he noted a general decrease in his mobility. He could not keep up with the beat when he danced, and his right lower limb was less flexible. His symptoms insidiously progressed until he noted a decreased ability to stop his forward motion when walking. He denied any tremor, drooling, or memory deficits. No limb or gait ataxia was noted. Motor examination revealed slight bradykinesis and rigidity. Head magnetic resonance imaging (MRI) and laboratory examinations revealed no abnormalities. He was diagnosed with Parkinsonism. The H-Y stage was 2.5. Levodopa benserazide (0.125 mg, tid) was prescribed with marked benefit. His daily activities were nearly normal. However, his symptoms progressed gradually, and by age 63 all of his movements had slowed significantly. Because of his son's illness, genetic testing was done and showed CAG abnormal repeats (35) (Table 1, Fig. 1). He was thus diagnosed with SCA2. At the age of 64, he could not walk or take care of himself. On physical examination he had a mask-like face, with forward flexion posture and his rigidity was significantly increased. He had a freezing gait (difficulty starting and turning). He was treated with levodopa benserazide (0.375 g tid) and amantadine (100 mg bid). His symptoms improved, and he was significantly better able to walk.

## 3 Discussion

SCA2 is an autosomal dominant disease caused by abnormal amplification of CAG (trinucleotide cytosine-adenine-guanine deoxyribonucleic acid) in the ATXN2 (12q23-q24.1) gene, which is diagnosed according to the criteria of SCA2.^[4]^ The usual range of CAG repeats is 11 to 31,^[5]^ with a pathogenic range of 34 to 200 repeats.^[2]^ ATXN2 mutations cause Parkinsonism,^[6]^ which are characteristic of bradykinesia, rigidity, tremor, etc according to the criteria of MDS.^[7]^ In 2007, Kim et al^[8]^ suggested that abnormal amplification of CAG in ATXN2 gene may be the Parkinsonism's causative mechanism. The report also pointed out that Parkinsonism is a clinical manifestation of SCA2 in the Chinese population, with amplification of CAG repeats between 33 and 47. In the first report of a Chinese family with SCA2 gene mutations and Parkinsonism, a family of four had 33 to 36 CAG amplifications.^[3]^ In a report from South Korea,^[8]^ 30 patients with ataxia had 38 to 51 CAG repeats, and among them 3 patients with Parkinsonism whose numbers of repeats were 32, 34, and 35. In a word, due to different CAG repeats, there are different clinical manifestations. And in SCA2 patients with Parkinsonism as a clinical manifestation, the frequency of abnormal CAG amplification is similar, with a lower repeat number ranging from 32 to 43.^[9–11]^ With ataxia as a clinical manifestation for SCA2 patients, there are a high range of CAG repeats between 32 and 79 times, some even up to 500.^[9]^ Our findings are consistent with those reported in the literature: the father, with Parkinsonism, which had 35 CAG repeats. the son, with ataxia syndrome, which had 43 CAG repeats. The molecular mechanisms underlying Parkinsonism caused by ATXN2 expansion remain unclear. Nevertheless, the report showed that repeating interruptions of somatic mosaicism from CAG stretch could result in Parkinsonism instead of ataxia.^[9]^

Our report showed that the Parkinsonism phenotype of SCA2 is responsive to levodopa. The study of Lu et al^[12]^ also illustrated this point.

In conclusion, the report showed that SCA2 can have different manifestations in the same family, the reason we consider is that it may be associated with the different repeat number of CAG. So, gene testing is needed when SCA2 is suspected even though there are different manifestations in the same family.

## Author contributions

**Data curation:** Ying Chang, Xiufeng Liu, Yanyan Li, Yayun Yan.

**Formal analysis:** Yuanyuan Li, Ying Chang, Yayun Yan.

**Funding acquisition:** Yuanyuan Li.

**Investigation:** Yuanyuan Li, Ying Chang, Yayun Yan.

**Methodology:** Yuanyuan Li.

**Project administration:** Yayun Yan.

**Resources:** Yuanyuan Li, Ying Chang.

**Software:** Ying Chang.

**Supervision:** Xiufeng Liu, Yanyan Li, Yayun Yan.

**Validation:** Ying Chang, Xiufeng Liu, Yanyan Li.

**Visualization:** Ying Chang, Xiufeng Liu, Yanyan Li.

**Writing – original draft:** Yuanyuan Li.

**Writing – review & editing:** Yuanyuan Li, Ying Chang, Yayun Yan.

ying chang orcid: 0000-0001-5383-5536.
